# Metagenomic Analysis of the Rhizosphere Soil Microbiome with Respect to Phytic Acid Utilization

**DOI:** 10.1264/jsme2.ME12181

**Published:** 2012-12-19

**Authors:** Yusuke Unno, Takuro Shinano

**Affiliations:** 1 NARO Hokkaido Agricultural Research Center, Hitsujigaoka 1, Toyohira, Sapporo, Hokkaido 062–8555, Japan

**Keywords:** inositol phosphates, metagenome, organic phosphorus, pyrosequence

## Abstract

While phytic acid is a major form of organic phosphate in many soils, plant utilization of phytic acid is normally limited; however, culture trials of *Lotus japonicus* using experimental field soil that had been managed without phosphate fertilizer for over 90 years showed significant usage of phytic acid applied to soil for growth and flowering and differences in the degree of growth, even in the same culture pot. To understand the key metabolic processes involved in soil phytic acid utilization, we analyzed rhizosphere soil microbial communities using molecular ecological approaches. Although molecular fingerprint analysis revealed changes in the rhizosphere soil microbial communities from bulk soil microbial community, no clear relationship between the microbiome composition and flowering status that might be related to phytic acid utilization of *L. japonicus* could be determined. However, metagenomic analysis revealed changes in the relative abundance of the classes *Bacteroidetes*, *Betaproteobacteria*, *Chlorobi*, *Dehalococcoidetes* and *Methanobacteria*, which include strains that potentially promote plant growth and phytic acid utilization, and some gene clusters relating to phytic acid utilization, such as alkaline phosphatase and citrate synthase, with the phytic acid utilization status of the plant. This study highlights phylogenetic and metabolic features of the microbial community of the *L. japonicus* rhizosphere and provides a basic understanding of how rhizosphere microbial communities affect the phytic acid status in soil.

As a constituent of indispensable biomolecules such as phospholipids, ATP and nucleic acids, phosphorus (P) is an essential element for crop production. Since P is frequently a limiting factor for crop growth and production, the application of P-containing fertilizer is necessary for the achievement of high, stable crop yields. World reserves of economically exploitable rock phosphate, the raw material of P fertilizers, are limited, and experts warn that the global stock will be depleted by half in 2050 and exhausted within the next century as a result of its intensive use for crop production (9, 42).

In soil, P is mostly present in forms not easily accessed by plants. Inorganic phosphate, an easily accessible form, is present at very low concentrations, typically approximately 1–10 μM in the soil solution (36). The low availability of inorganic P results from the high fixation capacity of the soil in which inorganic P tends to form insoluble salts with some cations, particularly aluminum, iron or calcium in inorganic and organic states (33). Up to 80% of total soil P is present in organic forms (48); however, to yield bioavailable P, inorganic P must be released from organic phosphates through mineralization processes mediated by phosphatases secreted from soil organisms and plant roots (*e.g.*, 33). P transformation from the organic to inorganic form greatly impacts the bioavailability of P in soil, and this process is highly influenced by various chemical and biological characteristics of soil, such as soil C, N, Al, Fe and Ca content, pH, and the presence of other plants and microbes in the soil (46). Therefore, the factors influencing the bioavailability of soil P are highly complex and require systematic analysis. *Myo*-inositol phosphates, particularly hexakisphosphates (such as phytic acid), which can constitute up to approximately 50% of organic P in soil (2, 30, 49, 50), have been researched as a means to improve the ability of plants to obtain P directly from phytic acid (1, 4, 16, 25, 35, 36, 44, 54). One of the apparent reasons for the low bioavailability of phytic acid is the low capacity of plant roots to secrete phytase, which releases plant-available inorganic P from phytic acid. On the other hand, phytic-acid-utilizing microbes are ubiquitous even in cultivated soils (21, 33, 52), so these microbes, especially those in rhizosphere soil, may be able to contribute to plant utilization of phytic acid from soil. Experimental evidence from various studies suggests that microbial processes such as mineralization, solubilization and desorption are important in soil P transformation (37), including the hydrolysis of phytic acid in the soil to yield a usable form of P (32).

The contribution of soil microbes to phytic acid availability in the soil was confirmed by inoculation trials in which various plant species were cultured in the presence of isolated phytic-acid-utilizing bacteria, resulting in significantly improved plant growth (19, 35, 52). In addition, we have often observed improved plant growth upon addition of phytic acid in pot trials, wherein *Lotus japonicus* was grown using long-term experimental field soil that was managed without P-containing fertilizers. Thus, in this study, we have attempted to elucidate the mechanism(s) by which plants interact with rhizosphere microbes, the bacterial community that converts soil phytic acid into a plant-available form of P.

The details of the microbial contribution to phytic acid utilization in soil are still unclear because this could require multiple biological functions, some of which are mentioned above. To better assess this complex, multifactorial microbial contribution, we conducted a comprehensive phylogenetic analysis with shotgun metagenomics using two molecular ecological tools: a) molecular fingerprint methods to compare ribosomal RNA gene diversity and b) high-throughput shotgun sequencing methods to determine the diversity of functional genes in soil microbial metagenomes. Whereas the molecular fingerprint approach readily permits analysis of phylogenetic diversity in multiple samples, the metagenomic approach yields an opportunity to generate more finely detailed taxonomic profiles and to estimate metabolic potential of microbial communities (14, 23, 40, 55).

In this study, we have attempted to utilize pyrosequencing technology on a rhizosphere soil sample and apply a comparative metagenomics approach to analyze the shift of the rhizosphere microbial community structure associated with phytic acid utilization. Such analysis enables the determination of the various microbial processes that play important roles in increasing the bioavailability of soil phytic acid to plants.

## Materials and Methods

### Sample preparation and sampling

Field soil was sampled from the long-term experimental field of Hokkaido University that has been managed with or without particular fertilizers since 1914; we collected soil from the no-P-fertilizer-applied plot on May 16^th^, 2009. In pilot study, we selected this soil from 3 soils tested by the serviceability of applied phytic acid to plants (data not shown). The soil, which contains low amounts of total P (approximately 1280 mg total P per kg soil), is classified according to USDA soil taxonomy as brown lowland soil of pH 6.2 (H_2_0) with cultivated soybean as the preceding crop. The field soil sample consisted of topsoil collected from ten randomly selected positions in the field; the samples were subsequently pooled to yield one sample. After sampling, soil was air-dried, sieved with a 2-mm mesh, mixed thoroughly, and stored in the dark at room temperature for use in subsequent experiments. Fukudo soil (commercial soil purchased from Hokkai Sankyo, Hokkaido, Japan) contains very low P (485 mg total P kg^−1^ soil) and very few microorganisms (data not shown) because of heat preprocessing. Field soil and Fukudo soil were mixed at a ratio of 1:20 (w/w), and approximately 1.6 kg of mixed soil was added to a 2-L pot (Table S1). The experiment consisted of the following two treatment conditions: a no-P-compound-applied pot and a phytic-acid-applied pot. In the latter treatment, soil was supplemented with phytic acid by addition of sodium phytate (Sigma Chemical Company, St Louis, MO, USA) to a concentration of 200 mg P kg^−1^ soil. In all treatment conditions, nitrogen (N) was applied at 150 mg N kg^−1^ soil with (NH_4_)_2_SO_4_, and potassium (K) was added at 150 mg K kg^−1^ soil with K_2_SO_4_. Fertilizers were separately mixed well into each pot a week before sowing. Each treatment had 11 pots: 10 for cultivation and one for obtaining soil without plants as bulk soil.

Seeds of *L. japonicus* MG20 (‘Miyakojima’), a model legume (39), were treated with concentrated sulfuric acid for 20 min and washed repeatedly with tap water (20), and then 10 seeds were sown per pot. Plants were cultivated in a glasshouse at Hokkaido University, and soil moisture content was maintained at approximately 60% with tap water. Two weeks after sowing, seedlings were thinned to five plants of similar size per pot. Cultivation experiments were carried out twice, from June 15^th^ to August 7^th^ (53 d) and July 19^th^ to September 6^th^ (49 d) in 2009. The range of average daily temperature during the experiment was 18–26°C. To avoid growth difference by lighting conditions, we changed the position every day during cultivation periods. At the end of cultivation, some plants grown with added phytic acid showed vigorous growth and had reached the flowering stage, while the growth of other plants was retarded and they did not flower at all. This phenomenon suggests that the flowering individuals had obtained more nutrients (*e.g.*, phosphorus) than those that did not. To distinguish their characteristics, plants that produced flowers were designated as Flowering (F), and those that did not as Not Flowering (NF) in this manuscript. The shoots were sampled for growth analysis and the roots were removed carefully from the pot to obtain rhizosphere soil. Rhizosphere soil was obtained by the following method: carefully obtained roots with rhizosphere soil were shaken vigorously in a 50-mL tube, and the fallen soil was designated as rhizosphere soil. Soil samples were frozen with liquid nitrogen and stored at −20°C for soil biome analysis. Part of the soil sample was lyophilized and analyzed for soil P content.

### Plant analysis

Shoots were sampled and oven-dried for more than a week at 65°C, and then dry weight and P content were determined. After dry weight was determined, the sample was digested with H_2_SO_4_–H_2_O_2_, and P content was determined by the molybdate blue method (28). Arbuscular mycorrhizal fungi colonization was checked in roots after partial digestion in KOH and staining with trypan blue (17).

### Soil P analysis

Samples of soil maintained in the presence or absence of phytic acid were collected at 0 d and 53 d during the 1st cultivation trial (from June 15^th^ to August 7^th^). Total soil P was determined by perchloric acid digestion (41), and plant-available phosphate was determined by Truog extraction (47). Analysis of Truog P and total soil P was performed in triplicate. For the analysis of soil phytic acid, phosphorus was extracted by shaking 1 g lyophilized soil at 200 rpm for 16 h at room temperature in 20 mL of a solution containing 0.25 M NaOH and 0.05 M Na_2_EDTA (ethylenediaminetetraacetate) (7). Extraction was carried out separately with 3 replicates. Extracts were centrifuged at 10,000 g for 30 min, then 7 mL of each extract was combined and 1 mL of a 49.8 μg P mL^−1^ water solution of methylene diphosphonicacid (MDPA) was added as an internal standard, frozen at −80°C, and then lyophilized. Approximately 100–200 mg of each freeze-dried extract was re-dissolved in 0.6 mL of a solution made by adding deuterium oxide (99.8% for NMR spectroscopy; Merck) to a solution containing 1.0 M NaOH and 0.1 M EDTA (1:9 by volume) and then transferred to a 5 mm NMR tube (N-5PL; Nippon Seimitsu Kagaku). Solution ^31^P NMR spectra were obtained using a JEOL alpha 600 spectrometer operating at 242.85 MHz for detection of ^31^P. Samples were analyzed using a 5.25 μs pulse width (45°), delay time of 2.0000 s, acquisition time of 0.4522 s, and broadband proton decoupling. The delay time used here allows sufficient spin-lattice relaxation between scans for P compounds in NaOH–EDTA (6). As suggested by Turner *et al.* (49), temperature was regulated at 20C to permit comparison between studies, reduce the number of scans required to obtain acceptable signals, and minimize temperature-associated degradation of compounds. Approximately 30,000 scans were acquired over the course of approximately 20.5 h. Chemical shift values were analyzed with respect to 85% H_3_PO_4_ and quantitatively determined by referring to a signal of MDPA (internal standard) observed in the sample solution (17.5 ppm). Spectra were plotted with line broadening of 2.21 Hz. Baseline correction was not carried out, and signal areas were calculated by integration. Concentrations of phytate were determined by adding areas of four signals arising at higher magnetic fields than those of inorganic phosphate in the ratio of 1:2:2:1 and compared with the area of the signal corresponding to MDPA.

### Soil biome analysis

Bulk and rhizosphere soil samples were harvested for the analysis of rhizosphere soil biomes. Rhizosphere and bulk soils, 0.5 and 5 g, respectively, were put directly into a beads tube (included in the ISOIL for Beads Beating kit; NIPPON GENE, Tokyo, Japan) in liquid nitrogen. Samples were treated by shaking for 30 s at 1/30 s with a MM300 mixer mill (Retsch, Tokyo, Japan). Soil DNA was then extracted using ISOIL (NIPPON GENE) according to the manufacturer’s instructions. For pyrosequencing analysis, the extracted DNA was further purified using CL-4B columns (GE Healthcare, Little Chalfont, UK) with 0.2% (w/v) polyvinyl polypyrrolidone (Sigma Chemical) (24). The DNA concentration was determined with a Nanodrop spectrophotometer (ND-1000; Nanodrop Technologies, Wilmington, DE, USA). For molecular fingerprint analysis, three each of microbiome DNA from F and NF rhizosphere soils were used. The V3 region of the 16S rDNA gene was amplified from soil DNA with the primer pair of GC341F and 518R (29). The following thermocycling program was used for PCR: (300 s at 95°C) × 1 cycle; (30 s at 94°C, 30 s at 65°C [−1.0°C cycle^−1^], 60 s at 72°C) × 10 cycles; (30 s at 92°C, 30 s at 55°C, 60 s at 72°C) × 25 cycles; and (120 s at 72°C) × 1 cycle, using Thermal Cycler Dice (TaKaRa Bio, Otsu, Japan). Each amplification reaction consisted of 200 nM of each primer, 10 ng template DNA, 1.25 U ExTaq DNA polymerase (TaKaRa Bio) and the manufacturer’s recommended buffer conditions. PCR products were examined by standard 1.5% (w/v) agarose 1× TAE gel electrophoresis with SYBR Green I staining to confirm product integrity. DNA concentrations of PCR products were measured with Quant-it DNA BR (Invitrogen, Carlsbad, CA, USA) according to the manufacturer’s instructions. Approximately 0.5 mg of PCR product was used for Denaturing Gradient Gel Electrophoresis (DGGE) analysis. Denaturant gel (35% and 60%) was made by mixing 0% and 100% denaturing stock solution containing 10% acrylamide, 150 μL of 10% ammonium persulfate and 15 μL tetramethylethylenediamine with each solution. Electrophoresis was performed at a constant voltage of 150 V and a temperature of 60°C with 1× TAE buffer for 5 h using the D-Code universal mutation system (Bio-Rad Laboratories, Hercules, CA, USA). DGGE Marker II (NIPPON GENE) was also applied to lanes at each end of the gel. After electrophoresis, gels were stained with SYBR Green I, and the bands were visualized with a LumiVisonPRO 400EX (Taitec, Tokyo, Japan).

For metagenomic analysis, two categories of microbiome DNA from F and NF rhizosphere soils were pooled (*n*=5), sequenced using the pyrosequencing method and compared to the functional genes in the SEED platform. The DNA was precipitated with ethanol and resuspended in water at a concentration of approximately 1 μg μL^−1^. All metagenome libraries consisted of approximately 5 μg DNA. Sequencing was performed using the Genome Sequencer-FLX system (454 Life Sciences, CT, US) with multiplex identifier tags (AGCACTGTAG and ATCAGACACG) from Dragon Genomics (TaKaRa Bio). Sequence reads were sorted into each library using tag sequences and screened to remove exactly matched sequences that are known to be either artifacts of the pyrosequencing approach using 454 sequencing system software or accessions in the *L. japonicus* Genome DB (http://www.kazusa.or.jp/lotus/). The DNA sequences were then analyzed in the metagenomics RAST pipeline (Version 3.1, http://metagenomics.theseed.org/) with the SEED platform (http://www.theseed.org/) using the NCBI BLASTX algorithm on the NMPDR server (Argonne National Laboratory; http://www.nmpdr.org/) (26). Every metagenome was compared to exactly the same data set using the SEED subsystems, as calculated by identifying matches to the SEED platform where a) the matched protein was curated to be in a subsystem, b) the e-value cut-off for the BLAST search was 1 × 10^−5^ and c) the minimum alignment length was 50 nt, according to the metagenomics RAST pipeline recommendations. The SEED arranges metabolic pathways into a hierarchical structure in which all genes required for a specific task are arranged into subsystems. At the highest level of organization, the subsystems include both catabolic and anabolic functions (*e.g.*, ‘Amino Acid and Derivatives’, ‘Carbohydrates’ and ‘Cell Division and Cell Cycle’) and at the lowest level, the subsystems represent specific pathways (*e.g.*, alkaline phosphatase, citrate synthase, and glycosyltransferase). All data are shown as the percentage of sequences showing similarities to known functions. The complete results of our sequence data analysis of *L. japonicus* rhizosphere soil microbiomes are provided as supplemental data.

### Statistical analysis

Data were analyzed statistically by one-way analysis of variance and Tukey’s test (HSD, threshold p<0.05) using SPSS 10.0 Software (SPSS, Chicago, IL, USA). DGGE bands matched in reference to DGGE Marker II (NIPPON GENE). DGGE profile comparisons were performed after signal normalization using Pearson’s similarity index, taking both band number and intensity into account (13). Unpaired group mean averages were used for dendrogram construction using the Quantity-one program (Bio-Rad). The statistical significance of the presence or absence of different metabolic subsystems in each metagenome was calculated using the STAMP (Statistical Analysis of Metagenomic Profiles) subsampling software (31).

## Results and Discussion

Total amounts of P and phytic acid in soil were significantly increased (p<0.05) by phytic acid application, while this application did not affect Troug P (Table 1). Phytic acid and inorganic P values in initial soil samples were similar to those observed in the samples after cultivation, regardless of phytic acid application. Furthermore, the analysis of soil phytic acid concentration using solution ^31^P NMR spectroscopy revealed that the addition of phytic acid to soil without plants did not cause conformational changes in other P compounds available to plants (Table 1). These results mean that applied phytic acid was maintained in a non-available P state in soil without plants. The availability of organic P in soil is generally considered to be determined by two major factors: a) the solubilization capacity of the soil, and b) phosphatase activity in the rhizosphere soil. Bacteria that can utilize soluble forms of phytic acid, such as is found in Na salts, are ubiquitous in soil (34), while those that can utilize phytic acid in scarcely soluble forms, such as Al- or Fe-phytic acid, are rare (52). Thus, it is thought that the utilization of soil phytic acid by microorganism(s) is inhibited mainly because free phytic acid eventually forms insoluble salts with Ca, Al and Fe on soil particles (18).

**Fig. 1:**
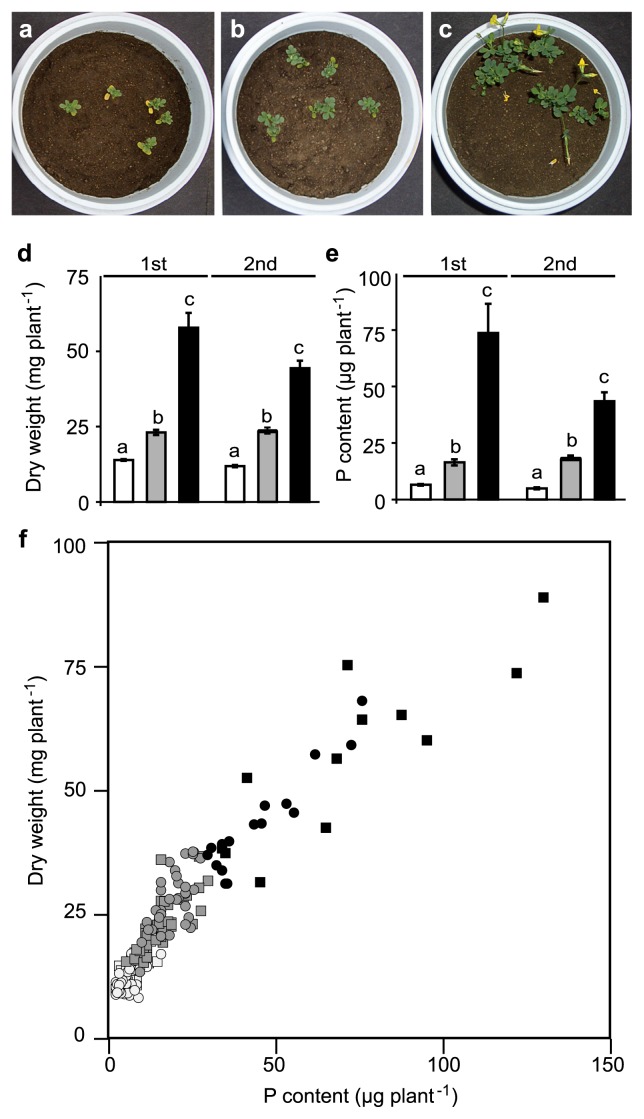
Results of *L. japonicus* growth 53 d (1st) and 49 d (2nd) after sowing, supplied with phytic acid or without added P. **a**. Seedlings grown with no P fertilizer added. **b** and **c**. Seedlings grown with phytic acid added. **d**. Shoot dry weight of seedlings in 1st and 2nd cultivations. **e**. P content of *L. japonicus* in 1st and 2nd cultivations. In **d** and **e**, Seedlings were grown in phytic acid-added pots with flowers (solid bars), without flowers (gray bars), or in no P-added pots (open bars), data are the mean ± SE, columns designated with different letters indicate significant differences by Tukey’s HSD test, *p* <0.05. **f**. Dry weight and P content of each seedling; 1st cultivation with flowers (solid circles) or without flowers (gray circles) in phytic acid-added pots, and no P-added pots (open circles); and 2nd cultivation with flowers (solid squares) and without flowers (gray squares) in phytic acid-added pots, and no P-added pots (open squares).

The growth of *L. japonicus* in pots with or without supplemental phytic acid is shown in Fig. 1. After 53 d or 49 d of growth in pots during the first and second cultivations, respectively, the dry weight and P uptake values of *L. japonicus* in the presence of supplemental phytic acid were significantly higher (p<0.05) than those in no-P-added plots in both cultivations (Fig. 1d and e). These results suggest that supplemental phytic acid is maintained in a non-available state in soil without plants, but transformed to a bioavailable state in rhizosphere soil. *L. japonicus* itself does not exhibit the ability to utilize phytic acid in aseptic conditions, and we cannot detect secretory phytase activity in these cultivation conditions (52). It is intriguing that all of the seedlings used in the current study utilized phytic acid during growth (Fig. 1). Furthermore, some plants grown with added phytic acid showed vigorous growth and reached the flowering stage, while other plants did not flower at all. The number of plants flowering in pots varied from 0 to 4 (Table S2). This suggests that the flowering individuals obtained more nutrients than those that did not. To distinguish their characteristics, plants that produced flowers were designated as Flowering (F) and those that did not as Not Flowering (NF). The F and NF plants were separated for subsequent analysis. The plants with phytic acid added to soil grew well, accumulated P in plants and reached the flowering stage (F; Fig. 1c), while others remained at the vegetative growth stage even with application of phytic acid (NF; Fig. 1b). Thus, one possible explanation for this effect was a cooperative interaction between *L. japonicus* roots and soil microorganisms that differed among individuals. The seedlings with flowers obtained more P than those that did not (Fig. 1f). Samples were categorized by flowering status to investigate how the different rhizosphere soil biomes influence tge phytic acid utilization status.

We assume that utilization of phytic acid as a P source might be a consequence of the presence and activity of soil microorganisms in the rhizosphere. It is possible that arbuscular mycorrhizal (AM) fungi in the soil may contribute to the utilization of phytic acid, as enhanced utilization of phytic acid by AM fungi colonization on wheat (*Triticum aestivum* L.) or red clover (*Trifolium pratense* L.) has been reported (15). Thus, the possibility of some contribution of AM fungi was considered in the present study; however, there was no observed infection of AM fungi on *L. japonicus* roots (data not shown), so it is unlikely that AM fungi had any effect under the present experimental conditions. In the rhizosphere, organic compounds are exuded from roots to the soil, where some of them, such as organic acid, phenolic compounds, and siderophores, modify nutrient availability through their capacity for chemical mobilization (*e.g.*, 56). Furthermore, we isolated phytic-acid-utilizing bacteria from the same field soil used in the current experiment, which fell into the betaproteobacteria; all most strains were identified as belonging to the *Burkholderia* subgroup, which promotes the growth of *L. japonicus* (52). Thus, we hypothesized that associative microorganisms, especially those bacteria which can supply available phosphorus from the decomposition of phytic acid to *L. japonicus*, are included in rhizosphere soil. It was assumed that these bacteria affect soil phytic acid availability via a cooperative relationship with the *L. japonicus* root.

**Fig. 2:**
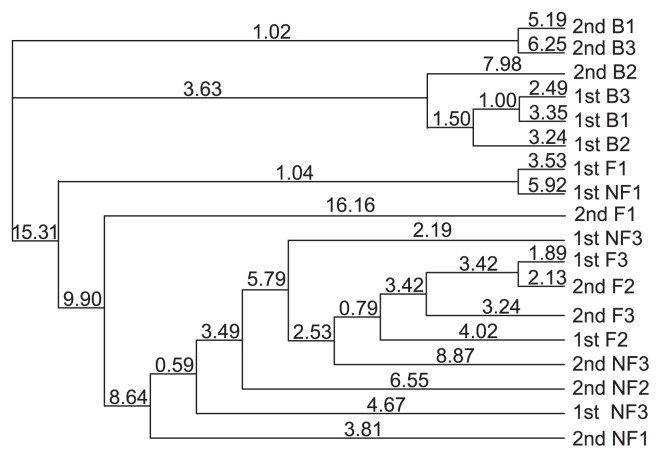
Corresponding cluster analysis for soil microbiome with different status; B as bulk soil, F as rhizosphere soil of *L. japonicus* with flowers, and NF without flowers.

PCR-DGGE analysis targeting the V3 region of 16S rDNA showed a clear difference in microbial diversity between bulk and rhizosphere soils; however, the microbial diversity of rhizosphere soil obtained from F and NF plants in pots with phytic acid applied could not be separated by cluster analysis (Figs. 2 and S1). This result indicated that while phylogenetic diversity in the rhizosphere soil microbiome changed in the presence of *L. japonicus* roots, there was no clear relationship between microbiome composition and phytic acid utilization intensity by *L. japonicus*, at least based on 16S rDNA analysis using DGGE. It has been reported that 16S rDNA diversity does not always relate to observable differences in physiological function, since parts of the microbial genome are hyper-variable (45). Unlike the 16S rDNA gene approach, the environmental shotgun sequencing approach permits the assessment of complete genetic information from a target microbial community (46, 51, 53). Accordingly, although we did not get a sufficient number of reads because of sample limitation, we analyzed the relative abundance of all genes and used the results of this analysis to generate a description of the functional potential of each community with massive parallel pyrosequencing systems.

To assess whether the phytic acid degradation status affected overall rhizosphere soil gene composition, we performed metagenomic profiling of *L. japonicus* rhizosphere soil microbiomes using a 454-FLX pyrosequencing system. A total of 12,244 reads passed the quality control filters, and these sequences were divided into two groups using multiplex identifier tags (8,445 reads derived from NF and 3,799 reads derived from F). Only 564 reads were similar to the *L. japonicus* genome (http://www.kazusa.or.jp/lotus/); therefore, our rhizosphere soil sampling methodology successfully eliminated plant genome contamination.

The remaining 11,680 reads, which did not yield significant hits against the *L. japonicus* genome, were used for global functional analysis using the SEED database to compare the two rhizosphere metagenomes. Unfortunately, many reads were excluded by our criteria, leaving the possibility of a functional gene, so 4,544 reads (38.9% of total) from both metagenomes, which were significantly similar to functional genes within the SEED, were used for taxonomic and functional analysis (Table 2). The taxonomic community profile derived by the assignment of protein-encoding genes showed that the majority of sequences belonged to bacteria and the difference between NF and F plants at the phylum level was not large (Table 3). This result was consistent with the results of molecular fingerprint analysis based on 16S rDNA diversity as mentioned above (Fig. 2). The most abundant phyla were *Proteobacteria* (43.0% of NF and 33.2% of F), *Fibrobacteres*/*Acidobacteria* (13.2% of NF and 19.6% of F), *Actinobacteria* (10.0% of NF and 12.6% of F) and the *Bacteroidetes*/*Chlorobi* group (10.25% of NF and 5.96% of F); these phyla were dominant in the *L. japonicus* rhizosphere in both F and NF plants; however, at class level, there was a considerable (>two-fold) shift in the abundance of five bacterial classes (*i.e.*, *Bacteroidetes* (class), *Betaproteobacteria*, *Chlorobi*, *Dehalococcoidetes* and *Methanobacteria*; Table S3). In addition, most fungi-like sequences belonged to *Ascomycota*; however, their ratio was quite low (0.51% of NF and 0.67% of F) and they differed only slightly.

The relative abundance of sequences assigned to each major subsystem in both soils was similar as judged from the relative distribution of the functional subsystems (Table 4). The most abundant subsystem category in the *L. japonicus* rhizosphere was ‘Carbohydrates’ (15.0% of NF and 13.5% of F), followed by ‘Amino Acids and Derivatives’ (9.1% of NF and 9.8% of F), ‘Protein Metabolism’ (7.2% of NF and 7.7% of F) and ‘Cofactors, Vitamins, Prosthetic Groups, Pigments’ (6.1% of NF and 6.9% of F). This indicates that similar pools of major genes with vital functions are present in both rhizosphere soil communities. The metabolic subsystem data were analyzed by the subsampling method to distinguish the differences between the two metagenomes at a 0.90 confidence level, and both ‘Glutamine, Glutamate, Aspartate and Asparagine Biosynthesis’ and ‘Glyoxylate Synthesis’ were found to be significantly increased in the phytic-acid-utilizing plant rhizosphere. Phytase (*myo*-inositol hexakisphosphate phosphohydrolase) belongs to a special class of phosphatases responsible for the dephosphorylation of phytic acid and has been found in a wide variety of organisms including animals, plants and microbes (10, 12, 43). Phytases in the phosphatase superfamily include histidine acid phosphatase, purple acid phosphatase, β-propeller phytase, protein tyrosine phosphatase and (PTP)-like myo-inositol polyphosphatases (8, 27, 32), although no phytases have been reported to be active in soil to our knowledge. However, we demonstrated that alkaline phosphatase genes, which have high degree of homology with the secreted alkaline phosphatase of *Aurantimonas* sp. SI85-9A1 (11), seemed to increase with increased availability of phytic acid in the rhizosphere soil (Tables 5, S4), so further research is necessary to determine whether any phosphatases are activated as phytase in soil. In addition, the abundance ratios of some gene clusters linked with phytate utilization, such as citrate synthase and malate synthase, changed with the phytic acid utilization status of the plant, while two metagenome samples showed similar global trends (Tables 5, S4). It is known that the release of organic acids into soil increases phytic acid solubility, making it more accessible to bacteria (17). As mentioned above, organic acids promote phytic acid degradation by phytase through the liberation of phytic acid from soil particles. Citrate synthase-like sequences in F sample have a high degree of homology with the citrate synthase gene of *Candidatus* Koribacter versatilis Ellin345 (YP_591351.1), which is a member of the phylum *Acidobacteria* originally isolated from pasture soil (38). Furthermore, the phylum of *Acidobacteria* is one of the abundant phyla and also had a more abundant ratio in F sample, so citrate synthase could participate in this process; however, few sequences were assigned to these functions, so further study is necessary.

In the analysis of functional genes, the category of secondary metabolism, including genes related to the production of antibiotic compounds and plant hormone-like compounds, accounts for a higher proportion in the F metagenome than that of the NF plants (4.0-fold, see Table 4). The rhizosphere is characterized by high microbial densities and the stimulation of microbial growth by roots (commonly known as the ‘rhizosphere effect’) caused by photosynthetic products exuded from roots (*e.g.*, sugars, organic acids, and polysaccharides). The quantity and quality of these root exudates are strongly influenced by both biotic and abiotic stresses, including the P status of the plant (*e.g.*, 56); as such, the shift of gene diversity in this study might reflect alterations in the quality and/or quantity of root exudates. On the other hand, we detected some gene clusters related to inositol metabolism, such as *myo*-inositol 2-dehydrogenase (EC 1.1.1.18), Epi-inositol hydrolase (EC 3.7.1.-) and *myo*-inositol-1 (or 4)-monophosphatase (EC 3.1.3.25). Not all phytase-producing bacteria can utilize inositol as a carbon source (33), but some show plant growth-promoting effects with phytate as the sole phosphorus and carbon source (52). Plant-growth-promoting rhizobacteria (PGPR) are a class of free-living bacteria in the rhizosphere that have helpful effects on plants (5, 22). The mechanisms proposed to explain PGPR effects are the production of plant-growth-regulating substances and the enhancement of nutrient availability (3, 5, 22). In addition, a large number of bacterial strains were identified as phytic acid degradative strains from the long-term experimental field, and some had high *L. japonicus* growth-promoting ability (52). It is thus conceivable that some bacterial strains, which have the ability to use phytate as a phosphorus and carbon source and other plant growth-promoting activity, might play a key role in phytic acid solubilization and decomposition in rhizosphere soil.

## Supplementary Material



## Figures and Tables

**Table 1:** P properties of two test soils

		Total soil P*	Truog P*	NaOH-EDTA extraction			

				Total P	IHP-P	Phosphate	Others
no P added	Initial	499 ± 22.8	26 ± 1.82	255	n.d.	144	111
	After	—	24 ± 2.48	260	n.d.	153	107
IHP added	Initial	681 ± 31.5	29 ± 2.11	447	169	151	127
	After	—	27 ± 3.14	437	171	145	121

Analyses of bulk soil P (mg P kg^−1^) change extracted in a solution containing 0.25 M NaOH and 50 mM Na_2_EDTA, with phosphorus detection by solution ^31^P NMR spectroscopy. Signals were quantified using an internal MDPA standard (see methods).

* Data are the mean ± SE of three samples.

**Table 2:** Pyrosequencing results

	NF	F
# of reads	8,445	3,799
Total read length	1,922,921	859,403
Average read length	227.7	226.2
# of unique reads	7,947	3,578
Matched in *L. japonicas* Genome		
# of reads	279	285
% of reads	3.3	7.5
Matched in SEED Non-redundant		
# of reads	3,322	1,222
% of reads	39.3	32.2
Matched in SEED Subsystems		
# of reads	1,955	702
% of reads	23.15	18.48

**Table 3:** Phylogenetic diversity (Phyla) in rhizosphere soil metage-nomes

Phylum	Relative abundance (%)		Ratio
	
	NF	F	F/NF
*Crenarchaeota*	1.67	1.77	1.06
*Euryarchaeota*	1.36	1.58	1.16
*Korarchaeota*	n.d.	0.19	—
*Actinobacteria*	10.01	12.57	1.26
*Aquificae*	0.07	n.d.	—
*Bacteroidetes/Chlorobi* group	10.25	5.96	−1.72
*Chlamydiae/Verrucomicrobia* group	0.61	0.84	1.38
*Chloroflexi*	3.44	4.19	1.22
*Cyanobacteria*	2.49	2.23	−1.12
*Deinococcus-Thermus*	0.24	0.37	1.54
*Fibrobacteres/Acidobacteria* group	13.24	19.55	1.48
*Firmicutes*	4.39	5.77	1.31
*Fusobacteria*	0.10	0.09	−1.11
*Planctomycetes*	2.11	1.86	−1.13
*Proteobacteria*	42.97	33.15	−1.30
*Spirochaetes*	0.17	0.09	−1.89
*Synergistetes*	0.17	0.19	1.12
*Thermotogae*	0.34	0.37	1.09
unclassified Bacteria	0.14	n.d.	—
Fungi/Metazoa group	2.76	4.75	1.72
*Viridiplantae*	2.69	4.28	1.59
dsDNA viruses, no RNA stage	0.10	n.d.	—

The occurrence of a phylum is shown as a percent of all phyla in each sample for two rhizosphere soil metagenomes. The difference in relative abundance between two rhizosphere soil metagenomes.

**Table 4:** Functional diversity based on SEED subsystems in rhizosphere soil metagenomes

Subsystem	Relative abundance (%)		Ratio
	
	NF	F	F/NF
Amino Acids and Derivatives	9.1	9.79	1.08
Carbohydrates	14.98	13.48	−1.11
Cell Division and Cell Cycle	1.79	1.61	−1.11
Cell Wall and Capsule	4.90	3.05	−1.61
Clustering-based subsystems	13.42	14.45	1.08
Cofactors, Vitamins, Prosthetic Groups, Pigments	6.11	6.90	1.13
DNA Metabolism	3.51	4.98	1.42
Fatty Acids and Lipids	1.50	0.64	−2.34
Macromolecular Synthesis	n.d.	0.16	—
Membrane Transport	1.67	1.77	1.06
Metabolism of Aromatic Compounds	1.96	1.28	−1.53
Miscellaneous	0.63	0.48	−1.31
Motility and Chemotaxis	1.96	2.57	1.31
Nitrogen Metabolism	0.81	1.12	1.38
Nucleosides and Nucleotides	2.30	3.21	1.40
Phosphorus Metabolism	1.73	1.77	1.02
Potassium metabolism	1.09	0.64	−1.70
Protein Metabolism	7.20	7.70	1.07
RNA Metabolism	2.71	3.37	1.24
Regulation and Cell signaling	1.79	2.57	1.44
Respiration	4.55	3.53	−1.29
Secondary Metabolism	0.12	0.48	4.00
Stress Response	3.57	3.05	−1.17
Sulfur Metabolism	2.02	1.61	−1.25
Unclassified	3.55	3.53	−1.01
Virulence	7.03	6.26	−1.12

The occurrence of subsystems is shown as a percent of all subsystems in each sample for the rhizosphere soil metagenomes. The difference in relative abundance between two rhizosphere soil metagenomes.

**Table 5:** Functional diversity based on SEED subsystems in rhizosphere soil metagenomes

	Relative abundance (%)		Ratio
		
	NF	F	F/NF
Outer membrane protein	0.05	0.27	5.42
Citrate synthase (si) (EC 2.3.3.1)	0.05	0.20	4.07
Leucyl-tRNA synthetase (EC 6.1.1.4)	0.05	0.20	4.07
putative integral membrane protein	0.05	0.20	4.07
Transcriptional regulator, TetR family	0.05	0.20	4.07
Glycosyltransferase (EC 2.4.1.-)	0.12	0.47	3.80
Alkaline phosphatase (EC 3.1.3.1)	0.07	0.27	3.62
ABC-type transport systems	0.10	0.33	3.39
Beta-galactosidase (EC 3.2.1.23)	0.25	0.07	−3.69
3-oxoacyl-[acyl-carrier protein] reductase	0.32	0.07	−4.79

Subsystems showing >3-fold ratio.
