# Supplementary material for: Metagenomic Analysis of the Rhizosphere Soil Microbiome with Respect to Phytic Acid Utilization
Source: Microbes Environ. 2012 Dec 19;28(1):120–7. doi: 10.1264/jsme2.ME12181 (PMC4070688; doi:10.1264/jsme2.ME12181)
Supplement: Supplementary file 1 [file 28_120_s1.pdf]

Supplemental information

Supplemental information contains following four files. Additional File 1 and 2 contain MID-tag removed sequence data.

Fig. S1. DGGE profiles of 16S rDNA fragments obtained by PCR amplification of DNA from bulk and rhizosphere soil of *L. japonicus.* Three samples on each treatment were investigated. Bulk as bulk soil, Rhizosphere as rhizosphere soil of *L. japonicus*, F as plant with flowers, NF as plant without flowers, and M as Marker.

Fig. S2. DGGE profiles of 16S rDNA fragments obtained by PCR amplification of DNA from bulk and rhizosphere soil of *L. japonicus.* Three samples on each treatment were investigated. 1st as 1st cultivation (53 d), 2nd as 2nd cultivation (49 d), Bulk as bulk soil, Rhizosphere as rhizosphere soil of *L. japonicus*, F as plant with flowers, NF as plant without flowers, and M as Marker.

Table S1. Properties of test soil before P treatments.

Table S2. Numbers of pots on phytic acid-added condition with the vigorous growth of L. japonicus with flowering.

Table S3. Phylogenetic diversity (Class) in the rhizosphere soil metagenomes (n. d., not detectable).

Table S4. Functional diversity based on the SEED subsystems in the rhizosphere soil metagenomes (n. d., not detectable).

Additional File 1. The fasta file (those ending .fa) from the F sample.

Additional File 2. The fasta file (those ending .fa) from the NF sample.

Fig. S1 Unno, Y

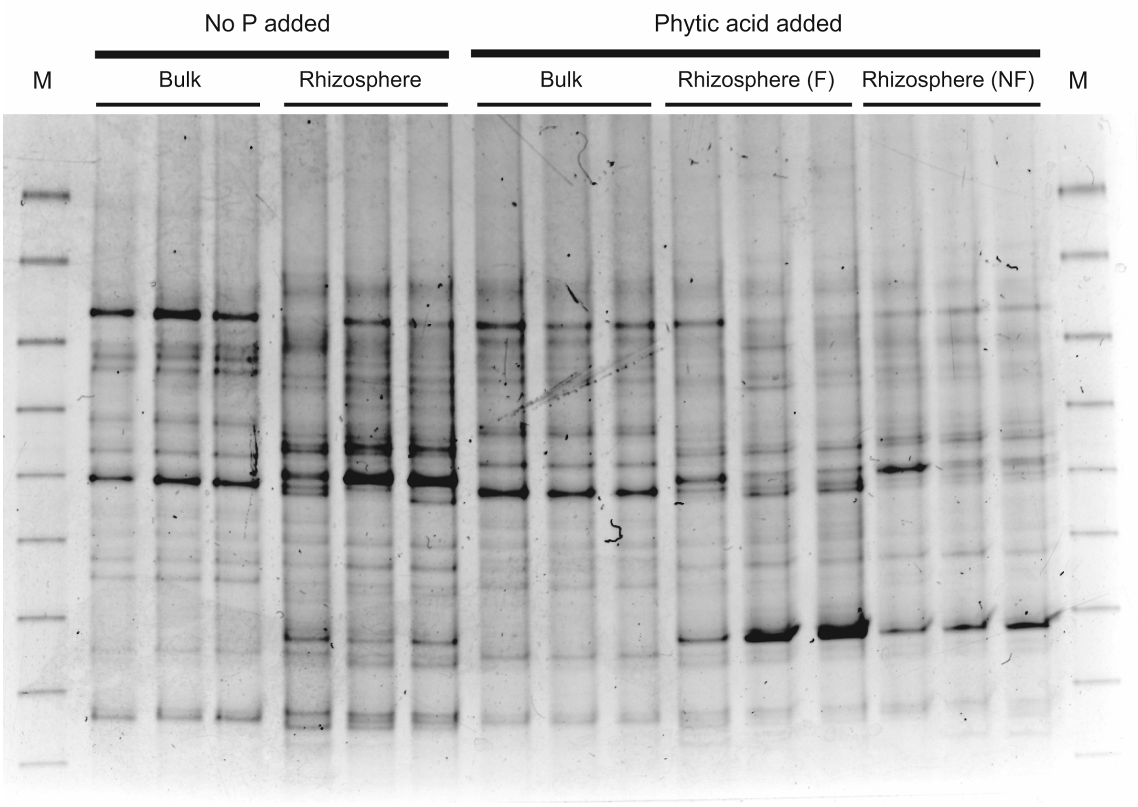
No P added
Phytic acid added
M
Bulk
Rhizosphere
Bulk
Rhizosphere (F)
Rhizosphere (NF)
M

Fig. S2 Unno, Y

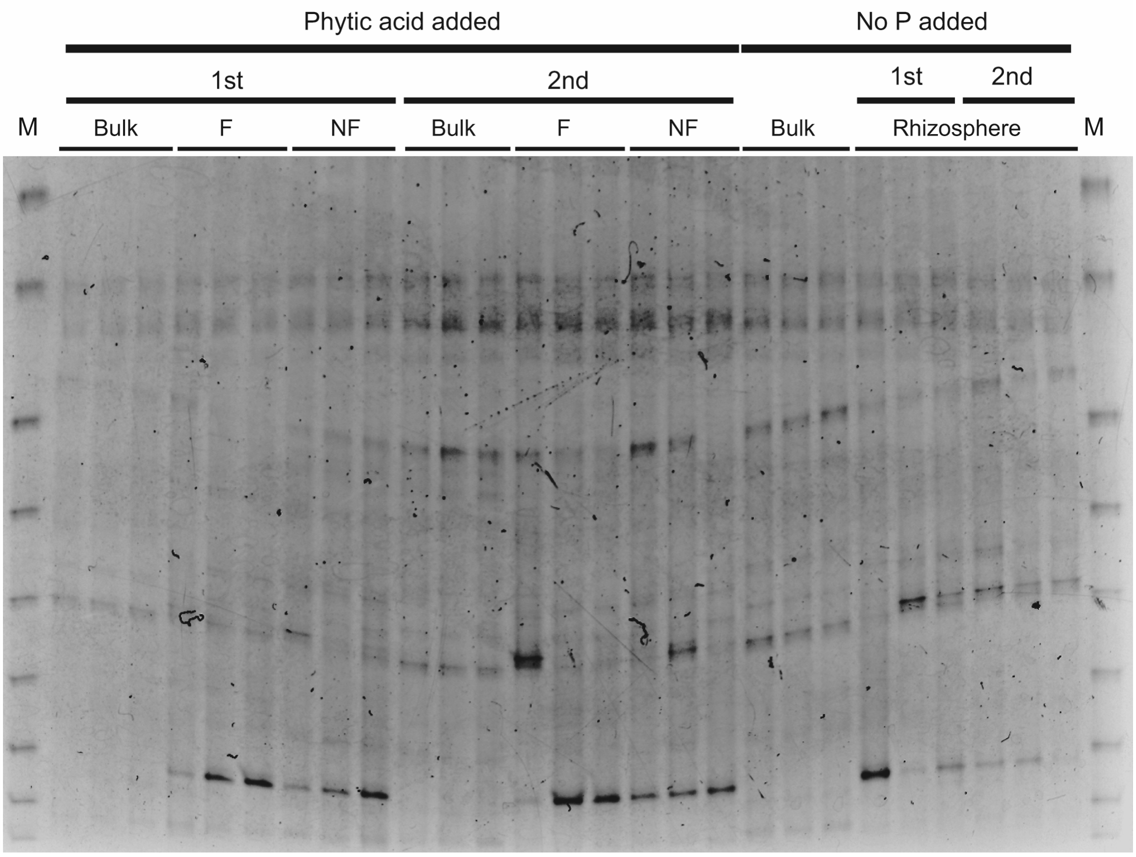
Phytic acid added
No P added
1st
2nd
1st
2nd
M
Bulk
F
NF
Bulk
F
NF
Bulk
Rhizosphere
M

Table S1.

pH(H2O)	Exch. K (mg K2O/100g soil)	Exch. Mg (mg MgO/g soil)	Exch. Ca (mg CaO/100g soil)	T-N (mg/g)
5.88 ±0.77	85.06 ±4.70	35.68 ±2.71	340.42 ±18.30	2.09 ±0.07

Table S2.

Flowering plants	Cultivation period	
	June 15th to August 7th	July 19th to September 6th
5	0	0
4	2	1
3	1	1
2	0	2
1	1	5
0	6	1

Table S3.

Class	Relative abundance (%) NF	F	Ratio F/NF
Acidobacteria	13.2	19.6	1.5
Actinobacteria (class)	10.0	12.6	1.3
Alphaproteobacteria	17.7	16.2	-1.1
Aquificae (class)	0.1	n.d.	-
Bacilli	1.9	2.9	1.5
Bacteroidetes (class)	12.6	6.1	-2.1
Betaproteobacteria	12.6	5.9	-2.1
candidate division TG1	0.1	n.d.	-
Candidatus Korarchaeum	n.d.	0.2	-
Caudovirales	0.1	n.d.	-
Chlamydiae	0.1	0.2	1.4
Chlorobi	0.4	0.8	2.3
Chloroflexi (class)	3.2	3.7	1.2
Chlorophyta	0.0	n.d.	-
Chroococcales	0.6	0.7	1.3
Clostridia	2.5	2.9	1.2
Dehalococcoidetes	0.2	0.5	2.4
Deinococci	0.2	0.4	1.5
delta/epsilon subdivisions	5.8	5.0	-1.2
Fungi	2.0	3.8	1.9
Fusobacteria (class)	0.1	0.1	-1.1
Gammaproteobacteria	6.8	6.1	-1.1
Gloeobacteria	0.5	0.7	1.2
Halobacteria	0.1	0.1	-1.6
Metazoa	0.7	0.9	1.3
Methanobacteria	0.1	0.3	2.8
Methanococci	0.1	0.1	-1.6
Methanomicrobia	0.8	1.0	1.3
Mollicutes	0.1	n.d.	-
Nostocales	1.0	0.6	-1.8
Oscillatoriales	0.2	0.3	1.6
Planctomycetacia	2.1	1.9	-1.1
Prochlorales	0.2	n.d.	-
Spirochaetes (class)	0.2	0.1	-1.9
Streptophyta	2.7	4.3	1.6
Syntrophomonadaceae	0.2	0.2	1.1
Thermococci	0.2	n.d.	-
Thermoplasmata	0.0	0.1	-
Thermoprotei	1.7	1.8	1.1
Thermotogae (class)	0.3	0.4	1.2
unclassified Proteobacteria	0.1	n.d.	-
Verrucomicrobia	0.5	0.7	1.4

Table S4.

	Number of sequences		Relative abundance (%		Ratio
	NF	F	NF	F	F/NF
Outer membrane protein	2	4	0.05	0.27	5.42
Citrate synthase (si) (EC 2.3.3.1)	2	3	0.05	0.20	4.07
Leucyl-tRNA synthetase (EC 6.1.1.4)	2	3	0.05	0.20	4.07
putative integral membrane protein	2	3	0.05	0.20	4.07
Transcriptional regulator, TetR family	2	3	0.05	0.20	4.07
Glycosyltransferase (EC 2.4.1.-)	5	7	0.12	0.47	3.80
Alkaline phosphatase (EC 3.1.3.1)	3	4	0.07	0.27	3.62
ABC-type transport systems	4	5	0.10	0.33	3.39
Glucose-methanol-choline (GMC)	3	3	0.07	0.20	2.71
Glutamate synthase [NADPH] large chain	3	3	0.07	0.20	2.71
Membrane protein	3	3	0.07	0.20	2.71
NAD-specific glutamate dehydrogenase	3	3	0.07	0.20	2.71
DNA-directed RNA polymerase beta subunit	7	7	0.17	0.47	2.71
Asparagine synthetase [glutamine-hydrolyzing]	4	4	0.10	0.30	2.71
Cysteine desulfurase (EC 2.8.1.7)	4	4	0.10	0.27	2.71
Malto-oligosyltrehalose trehalohydrolase	4	4	0.10	0.27	2.71
Acid phosphatase	1	1	0.02	0.07	2.71
2-oxoglutarate oxidoreductase, alpha subunit	2	2	0.05	0.13	2.71
putative 22 kDa kafirin cluster; Ty3-Gypsy	7	6	0.17	0.40	2.32
membrane protein, putative	5	4	0.12	0.27	2.20
dTDP-glucose 4,6-dehydratase (EC 4.2.1.46)	4	3	0.10	0.20	2.03
UDP-glucose 4-epimerase (EC 5.1.3.2)	4	3	0.10	0.20	2.03
ABC transporter ATP-binding protein	7	5	0.17	0.33	1.94
Transcriptional regulator	10	7	0.25	0.47	1.90
Adenylate cyclase (EC 4.6.1.1)	6	4	0.15	0.27	1.81
Conserved hyperthetical protein	6	4	0.15	0.27	1.81
3-polyprenyl-4-hydroxybenzoate carboxy-lyase	3	2	0.07	0.13	1.81
4-hydroxy-3-methylbut-2-enyl diphosphate	3	2	0.07	0.13	1.81
ABC-type nitrate/sulfonate/bicarbonate	3	2	0.07	0.13	1.81
Amidophosphoribosyltransferase (EC 2.4.2.14)	3	2	0.07	0.13	1.81
Aspartate-semialdehyde dehydrogenase (EC	3	2	0.07	0.13	1.81
Carbamoyl-phosphate synthase large chain	3	2	0.07	0.13	1.81

Cell division protein ftsZ (EC 3.4.24.-)	3	2	0.07	0.13	1.81
polyprotein	3	2	0.07	0.13	1.81
pyoverdine efflux carrier and ATP binding	3	2	0.07	0.13	1.81
Ribosomal large subunit pseudouridine synthase	3	2	0.07	0.13	1.81
RNA polymerase sigma-70 factor, ECF subfamily	3	2	0.07	0.13	1.81
Sugar phosphate isomerases/epimerases	3	2	0.07	0.13	1.81
Transcription termination factor Rho	3	2	0.07	0.13	1.81
tRNA (Guanine37-N1) -methyltransferase	3	2	0.07	0.13	1.81
putative retroelement	19	12	0.47	0.80	1.71
Oxidoreductase	7	4	0.17	0.27	1.55
Type I restriction-modification system	7	4	0.17	0.27	1.55
Putative polyprotein	26	13	0.64	0.87	1.36
Enoyl-CoA hydratase (EC 4.2.1.17)	6	3	0.15	0.20	1.36
predicted protein	6	3	0.15	0.20	1.36
Amino acid permease	8	4	0.20	0.27	1.36
Assimilatory nitrate reductase large subunit	4	2	0.10	0.13	1.36
cAMP-binding proteins - catabolite gene	4	2	0.10	0.13	1.36
Catalase (EC 1.11.1.6) / Peroxidase	4	2	0.10	0.13	1.36
Excinuclease ABC subunit A	4	2	0.10	0.13	1.36
Integral membrane protein	4	2	0.10	0.13	1.36
Manganese transport protein MntH	4	2	0.10	0.13	1.36
Miscellaneous; Unknown	4	2	0.10	0.13	1.36
Prolipoprotein diacylglyceryl transferase	4	2	0.10	0.13	1.36
PUTATIVE TRANSPOSASE	4	2	0.10	0.13	1.36
Quino(hemo)protein alcohol dehydrogenase	4	2	0.10	0.13	1.36
UDP-N-acetylmuramoylalanyl-D-glutamate--2,6-diaminopimelate lig	4	2	0.10	0.13	1.36
unknown	4	2	0.10	0.13	1.36
Oar protein	18	8	0.44	0.53	1.21
Cobalt-zinc-cadmium resistance protein czcA	9	4	0.22	0.27	1.21
Chaperone protein DnaK	5	2	0.12	0.13	1.08
ClpB protein	5	2	0.12	0.13	1.08
hypothetical protein-transmembrane prediction	5	2	0.12	0.13	1.08
Protein export cytoplasm protein SecA ATPase	5	2	0.12	0.13	1.08
tolB protein precursor, periplasmic protein	5	2	0.12	0.13	1.08
hypothetical protein	509	194	12.53	12.95	1.03

expressed protein	8	3	0.20	0.20	1.02
FOG: TPR repeat	8	3	0.20	0.20	1.02
putative gag-pol polyprotein	19	7	0.47	0.47	1.00
Alcohol dehydrogenase (EC 1.1.1.1)	6	2	0.15	0.13	-1.11
Aspartate aminotransferase (EC 2.6.1.1)	6	2	0.15	0.13	-1.11
NADH-ubiquinone oxidoreductase chain L	6	2	0.15	0.13	-1.11
Quinone oxidoreductase (EC 1.6.5.5)	6	2	0.15	0.13	-1.11
sensor histidine kinase	6	2	0.15	0.13	-1.11
two-component response regulator	6	2	0.15	0.13	-1.11
Transposase	23	7	0.57	0.47	-1.21
putative outer membrane protein, probably	14	4	0.34	0.27	-1.29
Glycosyl hydrolase, BNR repeat precursor	7	2	0.17	0.13	-1.29
Putative transcriptional regulator	7	2	0.17	0.13	-1.29
ABC transporter, ATP-binding protein	12	3	0.30	0.20	-1.48
DNA polymerase III alpha subunit (EC 2.7.7.7)	8	2	0.20	0.13	-1.48
Rhs family protein	8	2	0.20	0.13	-1.48
3-ketoacyl-CoA thiolase (EC 2.3.1.16)	4	1	0.10	0.07	-1.48
Ammonium transporter	4	1	0.10	0.07	-1.48
D-3-phosphoglycerate dehydrogenase	4	1	0.10	0.07	-1.48
Fe-S oxidoreductase	4	1	0.10	0.07	-1.48
Gamma-glutamyltranspeptidase (EC 2.3.2.2)	4	1	0.10	0.07	-1.48
Glycine dehydrogenase [decarboxylating]	4	1	0.10	0.07	-1.48
Oxidoreductase, Gfo/Idh/MocA family	4	1	0.10	0.07	-1.48
Phosphoglycerate kinase (EC 2.7.2.3)	4	1	0.10	0.07	-1.48
transcriptional regulatory protein	4	1	0.10	0.07	-1.48
Transposase and inactivated derivatives	4	1	0.10	0.07	-1.48
serine/threonine protein kinase	22	5	0.54	0.33	-1.62
ABC transporter, permease protein	9	2	0.22	0.13	-1.66
putative ABC transporter permease	9	2	0.22	0.13	-1.66
Cytochrome c oxidase polypeptide I	10	2	0.25	0.13	-1.84
Ribonucleotide reductase of class II (coenzyme	10	2	0.25	0.13	-1.84
Alanyl-tRNA synthetase (EC 6.1.1.7)	5	1	0.12	0.07	-1.84
Aminopeptidase	5	1	0.12	0.07	-1.84
Gamete-specific hydroxyproline-rich	5	1	0.12	0.07	-1.84
Integrase	5	1	0.12	0.07	-1.84

Phosphoribosylformylglycinamidine synthase	5	1	0.12	0.07	-1.84
putative oxidoreductase	5	1	0.12	0.07	-1.84
Succinyl-CoA ligase [ADP-forming] beta chain	5	1	0.12	0.07	-1.84
Thioredoxin reductase (EC 1.8.1.9)	5	1	0.12	0.07	-1.84
Tyrosine-protein kinase EpsD (EC 2.7.10.2)	5	1	0.12	0.07	-1.84
Glycosyltransferase	11	2	0.27	0.13	-2.03
Long-chain-fatty-acid--CoA ligase (EC 6.2.1.3)	11	2	0.27	0.13	-2.03
TonB-dependent receptor	17	3	0.42	0.20	-2.09
ABC transporter permease protein	6	1	0.15	0.07	-2.21
ATP-dependent DNA helicase UvrD/PcrA	6	1	0.15	0.07	-2.21
Cell division protein ftsH (EC 3.4.24.-)	6	1	0.15	0.07	-2.21
cell surface protein	6	1	0.15	0.07	-2.21
Excinuclease ABC subunit C	6	1	0.15	0.07	-2.21
Hydrolase (HAD superfamily)	6	1	0.15	0.07	-2.21
Signal transduction histidine kinase	6	1	0.15	0.07	-2.21
Streptococcal hemagglutinin protein	6	1	0.15	0.07	-2.21
transcriptional regulator, GntR family	6	1	0.15	0.07	-2.21
Two-component hybrid sensor and regulator	6	1	0.15	0.07	-2.21
Xanthine dehydrogenase, molybdenum binding	6	1	0.15	0.07	-2.21
putative membrane protein	7	1	0.17	0.07	-2.58
Sensory box histidine kinase/response	7	1	0.17	0.07	-2.58
unknown protein	15	2	0.37	0.13	-2.77
Beta-galactosidase (EC 3.2.1.23)	10	1	0.25	0.07	-3.69
3-oxoacyl-[acyl-carrier protein] reductase	13	1	0.32	0.07	-4.79
putative exported protein	11	n.d.	0.27	n.d.	-
ATP-dependent protease La (EC 3.4.21.53)	10	n.d.	0.25	n.d.	-
ATP-dependent Clp protease ATP-binding subunit	9	n.d.	0.22	n.d.	-
Glutamine synthetase type I (EC 6.3.1.2)	8	n.d.	0.20	n.d.	-
integrase/recombinase XerD	8	n.d.	0.20	n.d.	-
5-methyltetrahydrofolate-homocysteine	7	n.d.	0.17	n.d.	-
Lead, cadmium, zinc and mercury transporting	7	n.d.	0.17	n.d.	-
Permeases of the major facilitator superfamily	7	n.d.	0.17	n.d.	-
ATP-dependent RNA helicase	5	n.d.	0.12	n.d.	-
CAIB/BAIF family protein	5	n.d.	0.12	n.d.	-
Dehydrogenases with different specificities	5	n.d.	0.12	n.d.	-

Deoxyguanosinetriphosphate triphosphohydrolase	5	n.d.	0.12	n.d.	-
GTP-binding and nucleic acid-binding protein	5	n.d.	0.12	n.d.	-
Hypothetical transmembrane protein	5	n.d.	0.12	n.d.	-
LSU ribosomal protein L22p (L17e)	5	n.d.	0.12	n.d.	-
Maltodextrin glucosidase (EC 3.2.1.20)	5	n.d.	0.12	n.d.	-
Multiple sugar ABC transporter,	5	n.d.	0.12	n.d.	-
Probable Co/Zn/Cd efflux system membrane fusion	5	n.d.	0.12	n.d.	-
response regulator	5	n.d.	0.12	n.d.	-
